# Supplementary material for: Haematological predictors of poor outcome among COVID-19 patients admitted to an intensive care unit of a tertiary hospital in South Africa
Source: PLoS One. 2022 Nov 4;17(11):e0275832. doi: 10.1371/journal.pone.0275832 (PMC9635707; doi:10.1371/journal.pone.0275832)
Supplement: S1 Table — (DOCX) [file pone.0275832.s001.docx]

**S1: Multivariable analysis assessing the Association between gender, comorbidities on mortality among the COVID-19 patients admitted in ICU**

| **Characteristic** | **Adjusted RR (95% CI)** | **p-value** |
| --- | --- | --- |
| Gender: Female | 1.17 (1.01-1.35) | 0.036 |
| Hypertension | 0.96 (0.82-1.12) | 0.609 |
| Diabetes Mellitus | 1.03 (0.89-1.19) | 0.678 |
| Acute Kidney injury | 1.10 (0.91-1.33) | 0.328 |
| BMI | 1.14 (0.96-1.34) | 0.129 |
| Age at admission | 1.01 (1.01-1.02) | **<0.001** |
