# Supplementary material for: Haematological predictors of poor outcome among COVID-19 patients admitted to an intensive care unit of a tertiary hospital in South Africa
Source: PLoS One. 2022 Nov 4;17(11):e0275832. doi: 10.1371/journal.pone.0275832 (PMC9635707; doi:10.1371/journal.pone.0275832)
Supplement: S2 Table — (DOCX) [file pone.0275832.s002.docx]

**S2: Stratified analysis of gender by different age groups among the COVID-19 patients admitted in ICU**

| **Characteristics** | **Female (n=237)** | **Male (n=253)** | **p-value** |
| --- | --- | --- | --- |
| Age category 1 | | | 0.290 |
| <=30 | 12 (5.1%) | 7 (2.8%) |  |
| 31-50 | 82 (34.6%) | 99 (39.1%) |  |
| >50 | 143 (60.3%) | 147 (58.1%) |  |
| Age category 2 | | | 0.880 |
| <=40 | 35 (14.8%) | 40 (15.8%) |  |
| 41-50 | 59 (24.9%) | 66 (26.1%) |  |
| >50 | 143 (60.3%) | 147 (58.1%) |  |
| Age category 3 | | | 0.880 |
| <=40 | 35 (14.8%) | 40 (15.8%) |  |
| 31-60 | 142 (59.9%) | 146 (57.7%) |  |
| >60 | 60 (25.3%) | 67 (26.5%) |  |
| Age category 4 | | | 0.830 |
| <=40 | 35 (14.8%) | 40 (15.8%) |  |
| 41-55 | 101 (42.6%) | 101 (39.9%) |  |
| >55 | 101 (42.6%) | 112 (44.3%) |  |
