# Supplementary material for: Haematological predictors of poor outcome among COVID-19 patients admitted to an intensive care unit of a tertiary hospital in South Africa
Source: PLoS One. 2022 Nov 4;17(11):e0275832. doi: 10.1371/journal.pone.0275832 (PMC9635707; doi:10.1371/journal.pone.0275832)
Supplement: Attachment — Submitted filename: Response to reviewers comments -22122021.docx [file pone.0275832.s003.docx]

**Reviewers’ responses to reviewers**

**Title: Haematological predictors of poor outcome among COVID-19 patients admitted to an intensive care unit of a tertiary hospital in South Africa.**

**Response to Reviewer 1**

Thank you for your review of our paper and for the valuable recommendations. *We tried to make the introduction and discussion more concise. Changes are made in lines 94-96, 114-119, 131-132, 282-284, 292-295, 305 (manuscript with track changes).* We have answered each of your points below*.*

1. Have the authors measured the level of serum ferritin? if yes, how was it correlated with the disease severity?

***Response:*** *The serum ferritin was not measured in this study*

2. The authors have shown the ROC for CRP with the data not to be found anywhere. Can you please include these data in your manuscript?

***Response*:** *Thank you for highlighting this typing error. The ROC figure with CRP has been removed.*

3. Have the authors checked the Univariate and multivariate analysis of the identified risk factors?

***Response:*** Yes, both univariate and *multivariate analysis to identify risk factors were performed. Table 3 shows univariate (unadjusted) and multivariate (adjusted) analysis of risk factors.*

4. Generating a table with receiver operating characteristic analysis of promising markers for severity of COVID-19 may better clarify the article conclusion.

***Response:*** *We have provided a table of the ROC characteristic of the promising severity markers of COVID-19 (Table 4)*
