# Supplementary material for: Haematological predictors of poor outcome among COVID-19 patients admitted to an intensive care unit of a tertiary hospital in South Africa
Source: PLoS One. 2022 Nov 4;17(11):e0275832. doi: 10.1371/journal.pone.0275832 (PMC9635707; doi:10.1371/journal.pone.0275832)
Supplement: Attachment — Submitted filename: Response to Reviewers -PONE-D-21-33196.docx [file pone.0275832.s004.docx]

**Response to Reviewer #2**

Thank you for your review of our paper. We hereby give a point-by-point response to the comments provided.

**Comment 1:** The manuscript by Chapanduka et al, addresses in the critical care setting the role of blood tests to predict poor outcomes. Their finding concurs with previous observations in COVID-19. The study is well-designed and the analysis adequate. The manuscript is well-written. As a minor suggestion, a few early (and relevant) studies were not included or discussed where similar findings had already been reported (e.g., PMID: 32503668, PMID: 33549831, PMID: 32283162) and a meta-analyze was used instead (Ref #10). These should be incorporated and discussed in context, as the populations in those manuscripts had similarities and differences with the cohort presented in this manuscript

***Response 1:*** *Thank you very much, we highly value your recommendation. In the tracking manuscript line 304-326, The articles suggested were added and discussed in the manuscript as follows: “Liu et al., 2020 investigated the baseline NLR of 245 patients admitted to hospital with COVID-19 and found 8% higher risk of in-hospital mortality for each unit increase in NLR and 15-fold higher risk of death in patients with high NLR tertile [30]. However, in addition to high NLR, these patients were found to be older, more likely to be males, and have underlying comorbidities, compared with patients in the lowest tertile of NLR [30]. Núñez et al., 2020 investigated baseline characteristics of 282 hospitalised patients with COVID-19 and demonstrated that high NLR, at the moment of hospitalisation, predicts poor outcomes including death or critical care admission [31]. In contrast to our findings, they reported lymphopenia is a good independent predictor for ICU admission, mechanical ventilation, and death [31]. While the increased NLR was mostly due to the increase in neutrophils and lymphopenia was a constant finding in our ICU cohort, regardless of the outcome. Unlike our study which focuses on ICU patients with severe COVID-19, previous studies investigated the baseline NLR for patients at hospitalisation with variable COVID-19 severity [30,31]. On the other hand, Ma et al., 2020, investigated baseline characteristics for 81 patients with severe covid-19 and found a high NLR have higher incidence of ARDS and a higher rate of mechanical ventilation [32]. Comparably, analysis of leucocyte and neutrophil counts and the NLR in our ICU cohort, confirms the superiority of the NLR in the severe COVID-19 setting”.*

**Response to Reviewer #3**

Thank you for your review of our paper. Below is a point-by-point responses to the comments provided

**Comment 1:** The finding that was most significantly different from the mainstream results was the higher rate of female mortality, yet they do not address potential explanations for this. Could this be associated to other confounding external factors like obesity, or other demographic factors, pre-existing comorbidities or acute ones developed during the ICU admission?

***Response 2:*** *Thank you very much for the valuable comment. Interestingly, there were no confounding factors contributing to the higher mortality based on the available data. While this study focused on common risk factors of severe COVID-19 such as age, smoking status, and common comorbidities, other factors such as ethnicity and host genetic predisposition were not explored in this study. This study highlights the need for studies that focus on host genetic predisposition in African population.*

*In the revised manuscript, statistical analysis added to exclude confounding factor (lines 225-231 and Appendix-1,2) which states “Further analysis of the data, to elucidate the association of gender and comorbidities with mortality did not reveal any association between mortality and comorbid conditions namely, obesity, hypertension, diabetes mellitus and acute kidney injury (Appendix 1). Age was significantly higher among non-survival females (p<0.001) and further analysis was carried out to identify any gender differences between the various age categories. This analysis demonstrated no significant gender difference between the various age categories, as summarised in Appendix 2., and lines 408-417 paragraph has been added to the discussion which states: “In contrast to previous studies that demonstrated higher mortality among males, our study revealed a higher mortality among female patients [44, 45]. The description of the study sample by sex was similar. We conducted stratified analysis to assess the role of age and comorbidities on increased risk of death among female patients. No association identified that could explain female gender being at higher risk of mortality among COVID-19 patients admitted in the ICU. The comorbidities specifically analysed were obesity, hypertension, diabetes, and acute kidney injury. Advanced age had a statistically significant correlation with the risk of mortality in the study population in general regardless of gender (Table-1, Appendix-2). Considering that the Western Cape Province of South Africa has a unique ethnic profile, other possible risk factors such as host genetic predisposition need to be explored further [46].”*
